# Controlling Gaussian and mean curvatures at microscale by sublimation and condensation of smectic liquid crystals

**DOI:** 10.1038/ncomms10236

**Published:** 2016-01-04

**Authors:** Dae Seok Kim, Yun Jeong Cha, Mun Ho Kim, Oleg D. Lavrentovich, Dong Ki Yoon

**Affiliations:** 1 Graduate School of Nanoscience and Technology and KINC, Korea Advanced Institute of Science and Technology, Daejeon 305-701, Korea; 2 Department of Polymer Engineering, Pukyong National University, Busan 608-739, Korea; 3 Liquid Crystal Institute and Chemical Physics Interdisciplinary Program, Kent State University, Kent, Ohio 44242, USA

## Abstract

Soft materials with layered structure such as membranes, block copolymers and smectics exhibit intriguing morphologies with nontrivial curvatures. Here, we report restructuring the Gaussian and mean curvatures of smectic A films with free surface in the process of sintering, that is, reshaping at elevated temperatures. The pattern of alternating patches of negative, zero and positive mean curvature of the air–smectic interface has a profound effect on the rate of sublimation. As a result of sublimation, condensation and restructuring, initially equilibrium smectic films with negative and zero Gaussian curvature are transformed into structures with pronounced positive Gaussian curvature of layers packing, which are rare in the samples obtained by cooling from the isotropic melt. The observed relationship between the curvatures, bulk elastic behaviour and interfacial geometries in sintering of smectic liquid crystals might pave the way for new approaches to control soft morphologies at micron and submicron scales.

## Abstract (web summary)

The self-assembly of soft matter is sensitive to their morphology, which is changeable. Here, Kim *et al.* show how evaporation and condensation at high temperature modify the surface of a smectic liquid crystal film in a way controlled by the local Gaussian and mean curvature of its layered structure.

## Graphical abstract

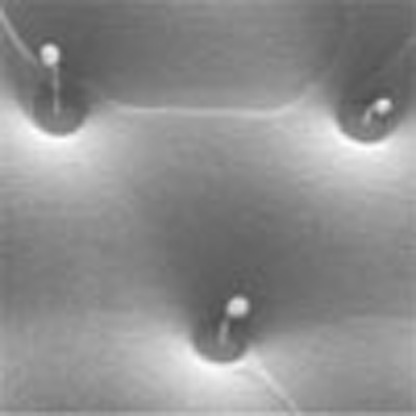

Morphogenesis, defined as the ensemble of mechanisms responsible for the formation of patterns and shapes, is a fundamental issue in natural sciences in general^1^ and physics of soft matter in particular. Especially profound examples of intimate relationship between the molecular structure of a soft material and its bulk curvatures and shapes are found in layered systems with a one-dimensionally periodic arrangement of molecules, such as block copolymers, stacked membranes and various liquid crystals. These materials show high flexibility and shape adaptation, controlled by equidistance of the layers and by balance of the interfacial and layers-bending energies^2^.

In the simplest realization, the so-called smectic A (SmA), the layer thickness is defined by the length of rod-like molecules. Smectics with soft boundaries, bordering either an isotropic fluid^3^,^4^,^5^,^6^,^7^,^8^,^9^,^10^,^11^ or air^12^,^13^,^14^,^15^,^16^,^17^,^18^, exhibit a very broad range of interesting morphologies. The well-known examples are highly irregular SmA nuclei emerging from the isotropic melt with layers bent into the arrays of focal conic domains^3^ and smectic films with free surface that exhibit non-flat interfaces with depressions^12^,^19^,^20^ and grooves^21^,^22^. Recently, new types of hierarchical assemblies have been added for SmA films with modulated substrates serving as topological templates^15^,^16^,^17^,^18^,^23^.

An intriguing question addressed in our work is how the shape of an SmA with a soft interface would change when the constituent molecules can leave the sample and re-condense on it. Such a system is a semifluorinated SmA with a free surface: the layers can be peeled off through sublimation at elevated temperatures^24^. From the studies of powdered metals and ceramics, it is known that sintering, defined broadly as a change in shape of a material held at elevated temperatures, is driven by the mean curvature of the interface that determines the local vapour pressure^25^,^26^. SmA features anisotropic surface tension and flexible layers that can bend.

We demonstrate that thermal treatment of SmA films leads to profound changes of their morphology, altering shape, mean and Gaussian curvature of the layers. The emerging structures are dramatically different from sintered crystalline and amorphous materials and from equilibrium SmA samples. The richness of the morphological changes is associated with a variable curvature of the SmA–air interface; this variable curvature mediates the local sublimation and condensation rates.

## Results

### Preparation and characterization of SmA films

**Figure 1:**
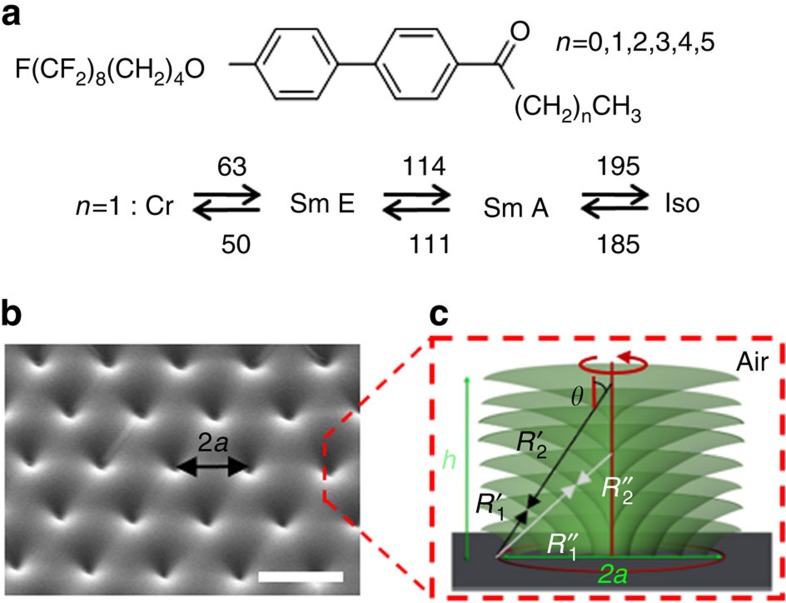
Materials used and SmA film with TFCDs array. (**a**) Chemical structure and phase diagram of semifluorinated **Y002**. (**b**) Equilibrium structure of a SmA film surface viewed under the SEM with a hexagonal array of indentations at the apexes of TFCDs. The scale bar is 10 μm. (**c**) TFCD structure; 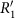 ,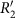, 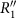 and 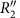 are the principal radii of layers curvature. The SmA molecules are oriented along the straight lines everywhere such as 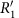

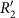
 and 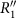

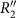
 that join the points of axial defect with the points of circular base. The structure is axially symmetrical, as indicated by the curved arrowed loop. The Gaussian curvature is negative everywhere; the mean curvature can be either positive (as for the point with the principal radii of curvature 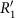 and 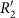) or negative (the point with 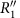 and 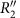). The angle *θ* is between the normal to the bottom substrate and the normal to the smectic layer (which is also the director).

The SmA film was prepared by placing a droplet of material Y002 (*n*=1), Fig. 1a, in its isotropic melted state onto a silicon substrate treated with polyethyleneimine (PEI) and then cooling it down below 195 °C into the smectic state, Supplementary Table 1. Y002 spreads into a flat film of thickness *h*=10–20 μm. The SmA molecules prefer to align parallel to PEI surface and perpendicularly to the free surface. SmA is in contact with air in all the experiments. Because of the antagonistic boundary conditions, the layers are curved in the vertical plane, with a radius of curvature *R*_1_ in Fig. 1c. To avoid wall defects, the layers are additionally curved in the horizontal plane, with a radius of curvature *R*_2_ originating at the axis of rotational symmetry in Fig. 1c. The folded layers form the so-called toroidal focal conic domain (TFCD). Figure 1c shows, as an example, two pairs of principal radii of curvature: the pair 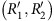 characterizes a point at a smectic layer that is close to the periphery of the TFCD, while 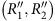 corresponds to a point close to the vertical axis of rotational symmetry of the TFCD. In each pair, the directions of the radii are antiparallel to each other; the radii originate at two linear defects, namely, at the horizontal circle of radius *a* (radii 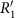 and 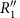), and at the vertical axial line (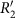 and 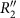). At the core of these two defects, the translational order of smectic layering is broken.

**Figure 2:**
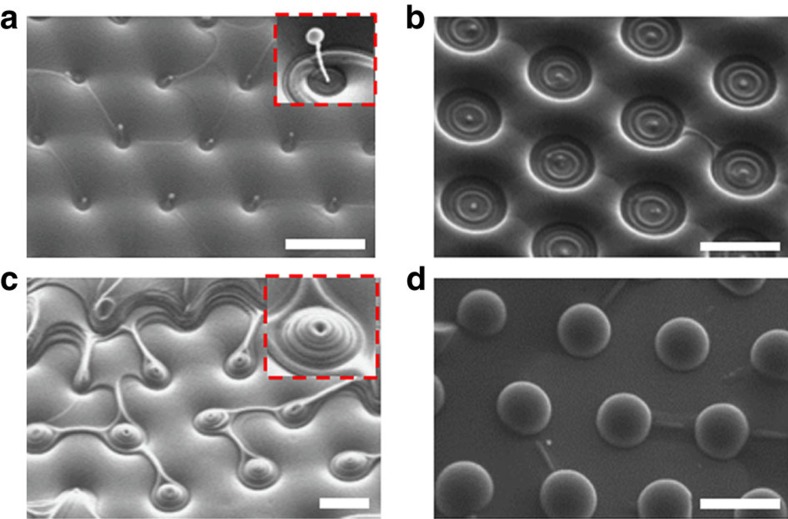
SEM images of SmA films transformed by sintering. (**a**) Udumbara flower-like morphologies produced by sintering at 130 °C for 120 h. (**b**) Concentric ring structures produced by sintering at 160 °C for 40 min and (**c**) at 180 °C for 8 min. (**d**) Domes produced by sintering at 190 °C for less than 2 min. All scales are 10 μm.

To summarize, within each TFCD, the layers are of a saddle-like shape with a negative Gaussian curvature, *G*=1/(*R*_1_*R*_2_)<0 (refs 12, 13, 14). Each TFCD is smoothly embedded into the surrounding system of flat horizontal layers, *G*=0, as the layers cross the cylindrical lateral boundary of the TFCD in a perpendicular fashion, Fig. 1c. The smectic–air interface has a non-flat profile with cusp-like depressions at the axial defect in the centre of each TFCD, Fig. 1b. When the smectic film with a hexagonal array of TFCDs is sintered at elevated temperatures within the range of stability of the SmA phase, it experiences dramatic restructuring, the result of which depends on the temperature and duration of sintering. Figure 2 shows examples of the most frequently met sintered structures.

### Sintering at 130 °C

A sample was kept at 130 °C for 120 h in air. Sublimation of the original film with a hexagonal array of indentations, Fig. 1b, results in partial removal of the smectic layers, Fig. 2a and Supplementary Fig. 1a,b. The edges of the remaining partial layers are seen in Fig. 2a as sharp bright curved lines connecting the interiors of the neighbouring TFCDs. An intriguing feature of the resulting morphology is an Udumbara flower-like formation, representing a vertical stalk centred at the symmetry axis of the original TFCD and capped by a tiny sphere (Fig. 2a). Other fluorinated SmA materials also show Udumbara flower-like structures, Supplementary Fig. 1c,d. The stack itself represents nested pieces of SmA layers, Supplementary Fig. 1c, or a linear array of toroidal and bead-like formations, Supplementary Fig. 1d. The region around the stalk, closer to the TFCD periphery, shows a much stronger degree of sublimation; removal of the material in this region is deeper than in the interstitial regions between the TFCDs with flat SmA layers^24^.

### Sintering at 160 °C

The sample was kept at 160 °C for 40 min. Sublimation removes most of the TFCDs and reveals the circular base of the domains, Fig. 2b, decorated by concentric rings. The rings are formed by many curved SmA layers, being much wider (100 nm–1 μm) than the single layer. The vertical cross-section of the ring (Supplementary Fig. 2) is close to a hemi-circle, the centre of which is slightly displaced below the substrate surface. The downward shift might be caused by the change of anchoring^27^ or by condensation of the material at the edges of the hemi-tori that would produce incomplete layers, Supplementary Fig. 2. If the sintering time is shorter than 40 min, then the removal of curved layers within the TFCD is only partial, similar to the case described below.

### Sintering at 180 °C

Sintering for about 6–10 min results in partial peeling of the layers. The layers are removed predominantly from the periphery of each TFCD and, to a somewhat lesser degree, from the interstitial regions between TFCDs, Fig. 2c, with a progressive loss of birefringence in the textures, Supplementary Fig. 3. As a result, the profile of the SmA film is formed by a hexagonal array of conical pyramids centred at the axes of the original TFCDs, Supplementary Fig. 4. Similar features of an accelerated sublimation at the periphery of TFCD and preserved central conical region are also evident in the textures reported previously^24^. The side surface of the conical pyramids exhibits hemi-tori, similar to rings observed in Fig. 2b and Supplementary Fig. 2, in which the concentric SmA layers curve to maintain equidistance and perpendicular orientation of molecules at the SmA–air interface.

### Sintering at 190 °C

Sintering near the melting point for about <2 min results in a very fast restructuring that generates dome-like structures (Fig. 2d). The film loses roughly 90% of its volume, since the average volume of each TFCD is on the order of 10^3^ μm^3^, while the volume of each dome is ∼10^2^ μm^3^, Supplementary Fig. 5. The domes have a circular base, resembling a portion of sphere (Fig. 2d, Supplementary Fig. 6c and Supplementary Fig. 7), although sometimes one also observes fat torus-like domes, with an indentation in the centre, Supplementary Fig. 6b. Atomic force microscope, Supplementary Fig. 7, and scanning electron microscope (SEM), Supplementary Fig. 5, shows that the domes are not exactly hemi-spherical. As in the case of rings, Fig. 2b, the reason might be a modified anchoring^27^ or capillary condensation at the edges of domes, Supplementary Fig. 5b.

### Sintering diagram of morphological transformations

**Figure 3:**
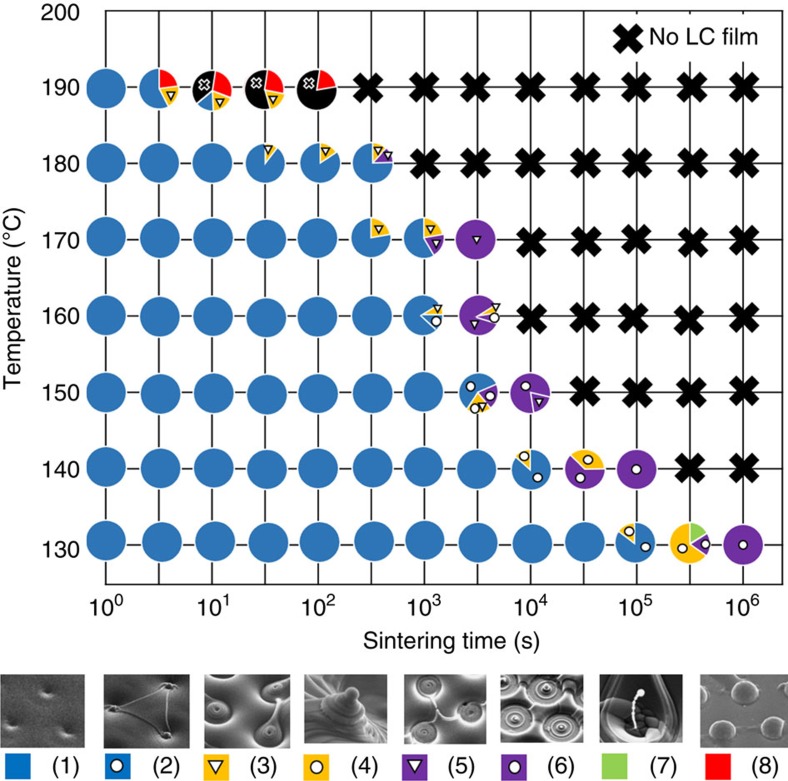
Sintering diagram of morphological transformations. Eight distinct types of resulting structures are identified as a function of the sintering temperature and duration: (1) TFCDs with cusp-like depressions at the axes; (2) TFCDs with spherical caps at the axes; (3) conical pyramids with cusp-like depressions at the apex; (4) conical pyramids with spherical caps; (5) concentric rings with central cusp-like depression; (6) concentric rings with spherical caps at the centre; (7) Udumbara flowers-like structures; (8) domes. In the diagram, the eight types are marked by coloured segments with the area approximately corresponding to the frequency of the observed structures for a given set of (temperature, time) conditions. The eight types are illustrated by the corresponding SEM textures.

**Figure 4:**
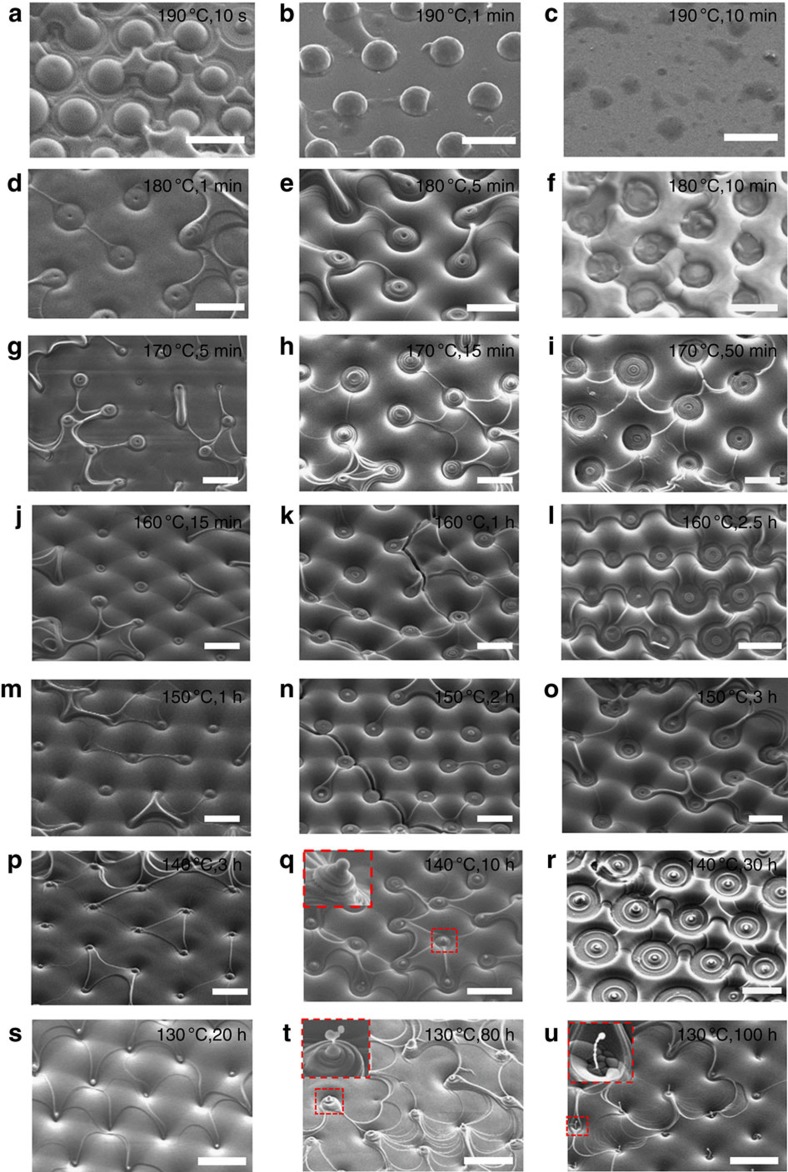
SEM images corresponding to the sintering diagram. (**a**–**c**) Sintering at 190 °C for 10 s, 1 and 10 min, respectively. (**d**–**f**) Sintering at 180 °C for 1, 5 and 10 min, respectively. (**g**–**i**) Sintering at 170 °C for 5, 15 and 50 min, respectively. (**j**–**l**) Sintering at 160 °C for 15 min, 1 and 2.5 h, respectively. (**m**–**o**) Sintering at 150 °C for 1, 2 and 3 h, respectively. (**p**–**r**) Sintering at 140 °C for 3, 10 and 30 h, respectively. (**s**–**u**) Sintering at 130 °C for 20, 80 and 100 h, respectively. All scale bars are 10 μm.

**Figure 5:**
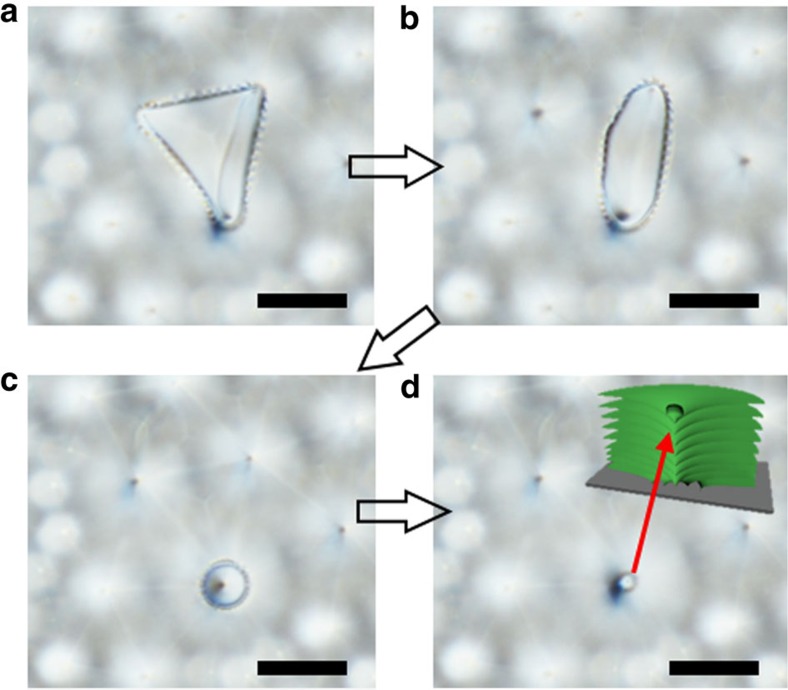
Spherical cap formation at the TFCD apex during thermal sintering at 130 °C (**a**) The uppermost layer of the liquid crystal ruptures, forming a shrinking island of a curvilinear triangular shape; (**b**) the ruptured layer shrinks; (**c**) the edge of the shrinking layer adopts a circular shape around the central cusp-like depression at the axis of the TFCD; (**d**) After ∼30 h, the shrinking layer is capped by a spherical surface. The inset in **d** is a schematic sketch of a cross-section of the TFCD with a spherical cap. Time interval between every two consecutive images is about 10 h. All scale bars are 10 μm.

To demonstrate the full set of scenarios and reproducibility of the results, the experiments were performed in the entire temperature range between 130 and 190 °C with a 10 °C increment, for different sintering times, ranging from 5 to 10^6^ s. For each temperature-duration condition, the experiments were performed at least 3–10 times, with qualitatively the same outcomes. The resulting diagram, Fig. 3 and Fig. 4, reveals eight distinct types of structures created by sintering:

1. TFCDs with cusp-like depressions at the centre, Fig. 4m; these are not much different from the original hexagonal arrays of TFCDs, Fig.1b, and are observed when the sintering time is short and the sintering temperature is low;
2. TFCDs with spherical caps instead of the depressions formed at relatively low temperatures (130–160) °C and relatively short sintering times, Fig. 4n,m,p,s,t. Figure 5 illustrates the process of the cap formation. First, the uppermost layer of the material sublimates, ruptures and shrinks; its edge changes from a curvilinear triangle, Fig. 5a, to an oval, Fig. 5b, and then a circle, Fig. 5c. At the final stage, the shrinking layer closes up by acquiring a spherical cap, Fig. 5d, presumably via re-condensation of SmA molecules from the air and diffusion within the SmA bulk. Diffusion should be facilitated at the core of the central defect where the layered structure of SmA is broken.
3. Conical pyramids with cusp-like depressions at the centres (Fig. 2c, Fig. 4d–l) formed at high temperatures (150–190) °C and low-to-moderate sintering times;
4. Conical pyramids with spherical caps at the centres (Fig. 4q,t), formed at temperatures (130–150) °C, and low-to-moderate sintering times;
5. Concentric rings with cusp-like depressions in the centres (Fig. 4i,l,o), formed from the conical pyramids at high temperatures as the sintering time increases;
6. Concentric rings with spherical caps in the centres (Fig. 2b, Fig. 4r), formed from the conical pyramids at low temperatures as the sintering time increases;
7. Udumbara flowers (Figs 2a and 4u), formed at low temperatures after relatively long sintering between 10^5^ and 10^6^ s; further sintering results in disappearance of the Udumbara flowers and formation of concentric rings at the substrate, with the Udumbara stalk being reduced to a small cup, Fig. 3 and Supplementary Fig. 8.
8. Domes (Fig. 4a–c), formed at the highest temperatures of 190 °C.

### Formation of the Udumbara flower structure

**Figure 6:**
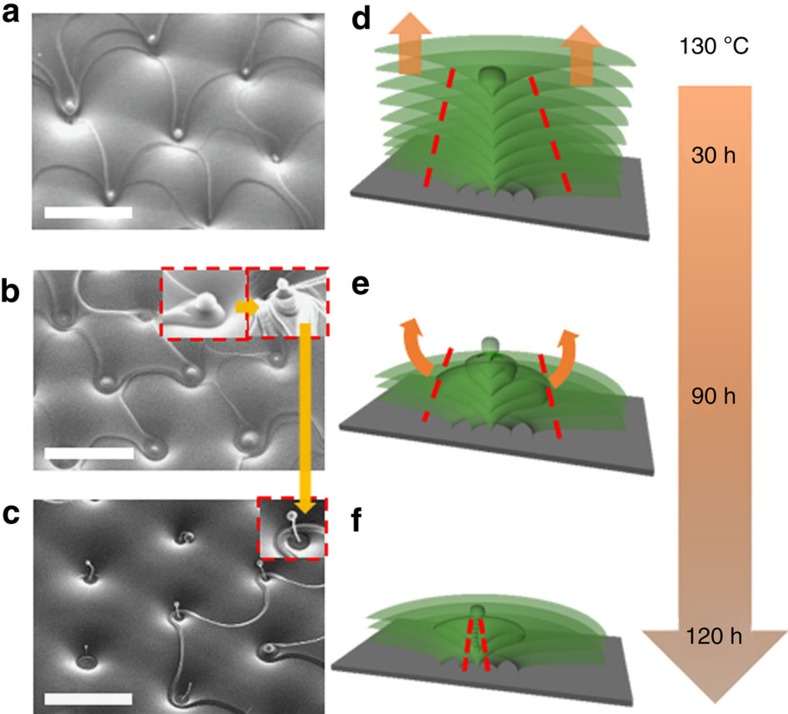
Formation of the Udumbara flower structure during sintering at 130 °C (**a**) After 30 h sintering, a spherical cap forms at the TFCD centre, following the process illustrated in Fig. 5. (**b**) Further sintering for ∼90 h removes more material from the periphery of TFCD, leaving conical pyramids at the central parts of TFCDs. (**c**) Udumbara structures formed after ∼120 h of sintering, with the stalks along the straight central defect lines of the original TFCDs. The insets in **b** and **c** are magnified SEM images, illustrating morphological change from the conical pyramid to the Udumbara structure. All scales are 10 μm. (**d**–**f**) Schematize layers configurations for the corresponding textures in **a**–**c**.

Figure 6 illustrates the development scenario of Udumbara flowers (7), as a function of sintering time. The first stage, Fig. 6a,d, is the formation of a spherical cap at the top of the vertical defect line, detailed in Fig. 5, through the rupture, shrinkage and closure of the uppermost layer of material. The uppermost layers around the central TFCD region continue to evaporate and shrink with time, leaving remnants of SmA layers around the central defect line; the result is a conical pyramid with a spherical cap at the apex, Fig. 6b. The side surface of the pyramid experiences reconstruction: tangential orientation of the molecules at the SmA–air interface associated with the exposed edges of the shrinking layers is being replaced by homeotropic orientation, which necessitates hemi-torical rings, as sketched in Fig. 6e. As time elapses, SmA molecules sublimate from both the areas around the pyramid and from the conical side surface of the pyramid, forming a stalk of the Udumbara flower with a spherical cap at the top and hemi-torical rings shrinking into small beads beneath it, Fig. 6c,f. If sintering extends beyond 120 h, the Udumbara flower vanishes. Generally, the universal patterns that form immediately before complete evaporation of the SmA films after prolonged sintering at moderate temperatures (130–180) °C, are of the types (5,6), with the concentric rings sharing the centre of the original TFCDs, Fig. 3 and Supplementary Fig. 8.

## Discussion

According to the Kelvin's equation, the saturated local vapour pressure over a patch of a deformed free surface is determined by its local mean curvature *H*:


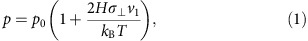


where *p*_0_ is the saturated vapour pressure over the flat surface, 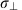 is the SmA–air surface tension for perpendicular anchoring, *v*_1_ is the volume of one molecule, *k*_B_ in the Boltzmann constant, *T* is the absolute temperature. Dependence of *p* on the mean curvature *H* is one of the key mechanisms responsible for the observed rich morphology of SmA shape reconstruction during sintering, since the local pressure determines the rate of sublimation/condensation, expressed as the number *n* of molecules that leave the surface per second per unit area, 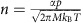; here *α* is the evaporation coefficient and *M* is the molecular weight.

The equilibrium SmA film with a free surface exhibits an array of TFCDs^12^,^19^,^28^ that satisfy the antagonistic boundary conditions. Packing of equidistant layers within each TFCD forms a cusp-like depression at the apex^12^,^14^,^19^,^29^. We adopt the simplest model of the interface by assuming that it follows the uppermost curved SmA layer; the assumption is justified by Fig. 1b, in which there are no edges that might be associated with the layer's ruptures.

**Figure 7:**
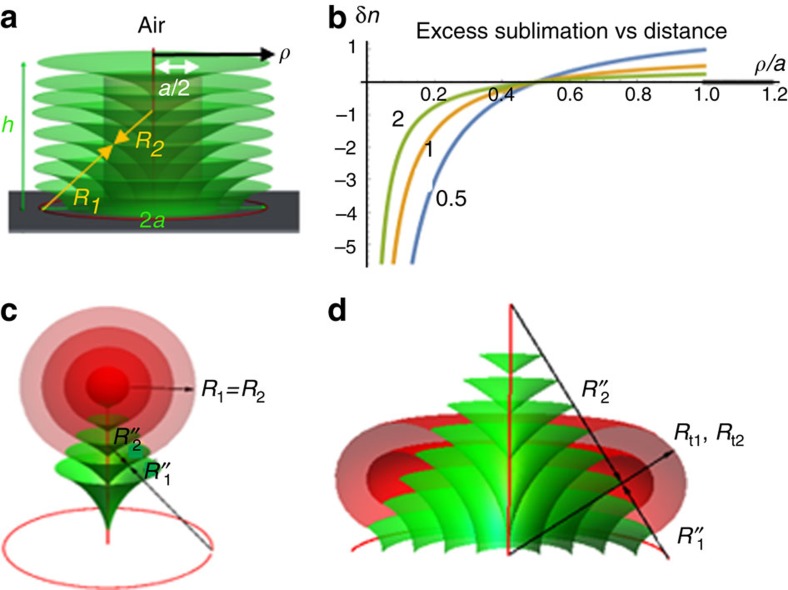
Morphological reconstructions during thermal sintering of smectic films (**a**) The mean curvature within the TFCD changes from negative inside the cylinder of radius *ρ*=*a/*2 to positive within the region *a/*2<*ρ*<*a*. (**b**) Excess sublimation rate (arbitrary units) versus normalized distance *ρ*/*a* from the TFCD axis, as predicted by equation (2), for three values of *h*/*a*=0.5; 1; 2 (shown on the plot); the region *ρ*/*a*>1 corresponds to the flat layers of zero curvature located in the interstitials between the TFCDs, with no excess sublimation; the region 0.5<*ρ*/*a*<1 corresponds to enhanced sublimation and the region 0<*ρ*/*a*<0.5 corresponds to enhanced condensation. (**c**) Smooth matching of equidistant layers between a TFCD remnant with *G*<0 and concentric spherical system with *G*>0. (**d**) Matching between a TFCD remnant with *G*<0 and a hemi-toroidal packing of equidistant SmA layers with *G*>0.

The SmA–air interface exhibits a variable mean curvature 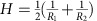 that depends on the distance *ρ* (measured along the bottom substrate from the TFCD axis) and the thickness *h* of the film (measured in the region between the TFCDs), Fig. 7a. The principal radii of the interface on top of a TFCD of a radius *a*, can be expressed in terms of *h* and *ρ*, as *R*_1_=*h*>0 and *R*_2_=*h*−*a*/sin*θ*, where *θ* is the angle between the normal to the substrate and the director, Fig. 1c. Therefore, the mean curvature 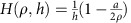 changes its sign as a function of *ρ*, being positive at the periphery, *a*/2<*ρ*≤*a*, and negative closer to the TFCD axis, 0<*ρ*<*a/*2. In the flat interstitial regions outside the TFCDs, *H*=0. According to the Kelvin's law, variation of the mean curvature produces a variation in the evaporation rates over the SmA–air interface:


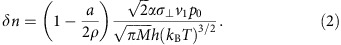


Emission of molecules is enhanced, *δn*>0, at the periphery of each TFCD, where *a*/2<*ρ*<*a*, and suppressed, *δn*<0, near the TFCD centre, at 0<*ρ*<*a/*2, Fig. 7b. In the flat interstitial regions between the TFCDs, *δn*=0. The regime *δn*<0 represents enhanced capillary condensation, as the lower local pressure drives the molecules emitted elsewhere, towards the TFCD centre. The axis of TFCD is the region of extremely favoured condensation, since for very small distances, *ρ*<<*a*, the negative mean curvature becomes very large, 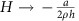.

Equations (1) and (2) are written in the limit of thermodynamic equilibrium, which is not the case of the experiment. Nevertheless, the model helps to understand the counterintuitive experimental features, most importantly, why the thermal treatment leaves the material in the TFCDs' centres and removes it from the TFCD periphery, eventually forming conical pyramids (3,4) and Udumbara flowers (7) centred at the TFCDs axes. Qualitatively, equation (2) predicts enhanced sublimation from the periphery of the original TFCDs, *a*/2<*ρ*<*a*, Fig. 7a,b, which explains the deep empty pockets around the Udumbara flower stalks, Fig. 2a and conical pyramids, Fig. 2c. Within the central regions, 0<*ρ*<*a*/2, Fig. 7a, equation (2) predicts condensation rather than emission, because of the negative mean curvature. In Fig. 7b, the range 0<*ρ*/*a*<0.5 corresponds to negative excess sublimation, that is, condensation. Note the dependence of the function *δn*(*ρ*) on the ratio *h*/*a* in Fig. 7b: in shallow films with a small *h*/*a*, evaporation from the periphery is stronger than in thicker films.

Variation of the local vapour pressure triggers material transfer along the smectic–air interface, supporting re-condensation of the emitted molecules at the central cusp-like depression of a TFCD, *ρ*<<*a*, where the negative mean curvature is very large. Condensation assists the formation of spherical caps in the structures of type (2), (4), (6–8), Figs 3, 4, 5, 6. The latter is expected to be a complicated dynamic process: Once the spherical cap of positive mean curvature is formed, the local emission rate increases, and the cap could evaporate, leaving a depression behind (structures (1), (3) and (5)). Experimental data suggest that the depressions are favoured at higher temperatures and the spherical caps at lower temperatures; in many cases, these two features coexist, see, for example, Fig. 4h, reflecting a fine balance of pressure gradients, emission/condensation rates, diffusion, surface anchoring and bulk elasticity.

Dynamics of spatially variable sublimation, condensation and restructuring through replacement of unfavourable tangential molecular orientation at the SmA–air interface with a perpendicular orientation implies that the film profile is constantly changing in space and, importantly, time. Consider the scenario in which a pyramid (3,4) is formed, Figs 2c and 6b. As the molecules leave the region *a*/2<*ρ*<*a*, there are two newly formed SmA–air interfaces. One is a cylindrical boundary between the original TFCD and the flat SmA layers outside it, of a radius *ρ*=*a*. The second newly formed surface is a cylinder of a smaller radius *ρ*≈(1/2)*a*, Fig. 7a. It encloses the central TFCD region that is a predecessor of the pyramids and Udumbara flowers. Once this cylindrical surface of radius *ρ*≈(1/2)*a* forms, it becomes the site of preferred emission, because of its positive mean curvature that increases from the initial value *H*=(1/*R*_1_+1/*R*_2_)/2*=*1/(2*ρ*)≈1/*a* to a larger value, as the material evaporates and the remaining central portion of the TFCD shrinks. Sublimation eventually forms a pyramid or an Udumbara stalk, exposing the peripheral parts of TFCD bases and leaving the remnants of SmA in the interstitial regions, where *H*=0 and *δn*=0. It is expected that some of the emitted molecules can re-condense, helping to produce the circular rings patterns of type (5) and (6), Fig. 2b. Since the sublimation progresses from the top to the bottom, it is natural to expect that for relatively short sintering times, the intermediate structure would be that of a central conical pyramid, Figs 2c and 6b (structures of type (3) and (4)).

The experiment demonstrates that in all reconstructed morphologies, the orientation of molecules remains perpendicular to the SmA–air interface. This is the case of hemi-toroidal formations of positive Gaussian curvature in Fig. 2b,c, and spheres in Fig. 2a,d. The SmA layers within the TFCDs, spherical and toroidal packings are all of the Dupin cyclides types^2^, with focal surfaces in the form of lines (for TFCDs and concentric tori) and points (for spheres). Matching between the remnants of TFCDs and spheres/toroids is facilitated by the geometrical rules according to which the normal vectors to the SmA layers are straight lines^2^ everywhere. For example, the normal lines in Fig. 1c join points of the axial defect to the points of the circular TFCD base. These rules are also observed in matching the TFCDs with the spherical packings, Fig. 7c, and toroidal layers, Fig. 7d. In Fig. 7c, the concentric spherical layers have a common centre of curvature at the TFCD apex. The layers from within the TFCD (shown in green) and the spherical (red) part match each other smoothly, as both families of layers are perpendicular to the conical interface separating them, Fig. 7c. This construction, proposed by Sethna and Kleman^30^ and confirmed for SmA droplets^31^, allows one to match layers with positive and negative Gaussian curvature. Similar consideration applies to toroidal formations, Fig. 7d. In Fig. 7d, the toroidal caps of a positive Gaussian curvature are deposited on the lateral conical surface; other cases, such as toroidal formations around the cylindrical Udumbara flower stalks, Fig. 2a, and relatively flat bases of TFCDs, Fig. 2c, follow the same basic geometry. The regions with a different Gaussian curvature are separated by interfaces that carry an elastic energy^32^. This energy, however, is much smaller than the anisotropic surface anchoring energy gain, as discussed below for the case of hemispherical domes.

The dome caps the circular base of the TFCD, thus replacing the surface energy 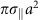 or 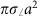 of the TFCD base exposed to air, with the energy 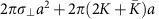. Here 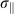, 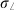 and 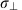 are the surface tensions for tangential, tilted and perpendicular orientation of molecules at the SmA–air interface, respectively, *K* is the splay elastic constant and 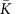 is the saddle splay elastic constant. The calculation of the elastic energy is based on the standard free energy density of SmA deformations,


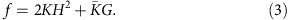


For the expected *σ*_⊥_=2 × 10^−2^ J m^−2^ (ref. 33), *a*=5 μm, *K*=30 pN (ref. 2), the surface energy of the hemispherical dome, 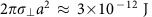, is significantly higher than its elastic energy, of the order of 4*πKa*≈10^−15^ J; although the saddle-splay modulus 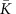 is not known for our system, it could not change the balance, as 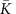 is unlikely to be orders of magnitude larger than *K* (ref. 2). The estimates above show that the anisotropic surface tension is a prevailing factor in shaping the SmA free surface. The surface tension coefficients for unfavourable tangential and tilted orientation should satisfy the condition 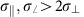 for the spherical domes to cap the TFCDs remnants. Although the surface tension of the free SmA surface has been measured only for the case of homeotropic anchoring^33^, it is very likely that the needed condition is satisfied. Tilted or tangential alignment of SmA molecules implies reconstruction of SmA layers through dislocations or through creation of a corrugated profile. For small tilts *α* from the perpendicular alignment, the surface tension increases as 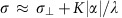, where *λ* is the length scale on the order of the SmA layer thickness^34^. For the typical *K*∼30 pN and *λ*∼3 nm, one estimates *K*/*λ*∼10^−2^ J m^−2^, which is of the same order as *σ*_⊥_=2 × 10^−2^ J m^−2^ (ref. 29). The conclusion about the prevalence of anisotropic surface anchoring energy gain 
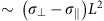
 over a patch of area ∼*L*^2^, as compared with the typical bulk energy cost, ∼*KL*, remain valid for other geometries of packing, as long as 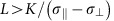, that is, practically for all scales exceeding the molecular length *λ*.

In summary, thermally sintered SmA films produce morphologies with spherical and toroidal elements with positive Gaussian curvature that is rarely met in equilibrium smectic samples. One of the reasons is that *G*>0 implies an increase of the mean curvature and thus a higher energy cost of packing. The complex morphological changes involve a number of contributing mechanisms, such as sublimation and condensation with the rates that vary in space and time, anisotropic interfacial energies, bulk elasticity and anisotropic diffusion of matter. We outlined how the classic Kelvin equation can contribute to understanding of the key observed features. Much more needs to be done, both experimentally and theoretically, before the morphological dynamics can be described accurately. However, it is already clear that sublimation/condensation of smectic liquid crystals represents a very intriguing field for research and potential applications in terms of submicron-scale manipulation of soft matter. The presented phenomena introduce a new concept in soft matter design that uses sublimation and condensation as the tool to control curvature of layers and interfaces. The theme is connected to the problem of morphogenesis in living matter. It would be of especial interest to combine the effects of thermal sublimation/condensation with the recently proposed approaches to topological templating^15^,^17^ that can control the density and geometry of focal conic domains.

## Methods

### Synthesis of sublimable liquid crystals

The LC materials, **Y001**–**Y006** were prepared as previously reported^19^. Phase transitions for the samples were characterized by differential scanning calorimetry (Q1000 V9.9 Build 303).

### Fabrication of morphologies by thermal sintering

Silicon wafers were cleaned using acetone and methanol, followed by rinsing with deionized water. The cleaned silicon substrates were spin-coated with PEI (Aldrich; MW: 60,000) for tangential anchoring of the LC. A crystalline powder of **Y002** at the silicon substrate was heated to the isotropic phase (200 °C) on a hot stage (LINKAM LTS350) regulated by a temperature controller (LINKAM TMS94). The sample was cooled to 190 °C at a rate of 10 °C min^−1^. The sublimation experiments were performed in the range between 190 and 130 °C with an increment of 10 °C for different sintering times, ranging from 5 to 10^6^ s. Thermal treatment was performed in the air. The instantaneous rates for mass loss percentage at the temperatures 190, 180, 160 and 130 °C, were determined to be 1.64, 0.82, 0.30 and 0.02% per °C, respectively, Supplementary Fig. 9. The volume available for the SmA molecules to sublimate is orders of magnitudes larger than the volume of the original SmA film (cubic decimetres versus cubic millimetres). Following the thermal treatment, the samples were cooled down to the room temperature, preserving the SmA structure (as verified by X-ray diffraction). The surface morphology was visualized by sputter-coating platinum (Pt) film and imaging the latter under a SEM.

### Imaging morphologies of thermally treated samples

The thermally annealed samples were coated with a 5 nm-thick layer of Pt and then observed by a field emission SEM (FE-SEM; Hitachi, S-4800) with 7 kV and 7 μA in high vacuum. Surface topography measurements were performed using an atomic force microscope (Bruker, Multimode-8) equipped with a 100 μm^2^ scanner in tapping mode under ambient conditions and an antimony doped silicon cantilever with a spring constant of 20∼80 N m^−1^ and frequency (*f*_0_) of 300 kHz was used.

## Additional information

**How to cite this article:** Kim, D. S. *et al.* Controlling Gaussian and mean curvatures at microscale by sublimation and condensation of smectic liquid crystals. *Nat. Commun.* 7:10236 doi: 10.1038/ncomms10236 (2016).

## Supplementary Material

Supplementary Information Supplementary Figures 1-9 and Supplementary Table 1
