# Supplementary material for: Controlling Gaussian and mean curvatures at microscale by sublimation and condensation of smectic liquid crystals
Source: Nat Commun. 2016 Jan 4;7:10236. doi: 10.1038/ncomms10236 (PMC4725769; doi:10.1038/ncomms10236)
Supplement: Supplementary Information — Supplementary Figures 1-9 and Supplementary Table 1 [file ncomms10236-s1.pdf]

Supplementary Figures

Supplementary Fig. 1

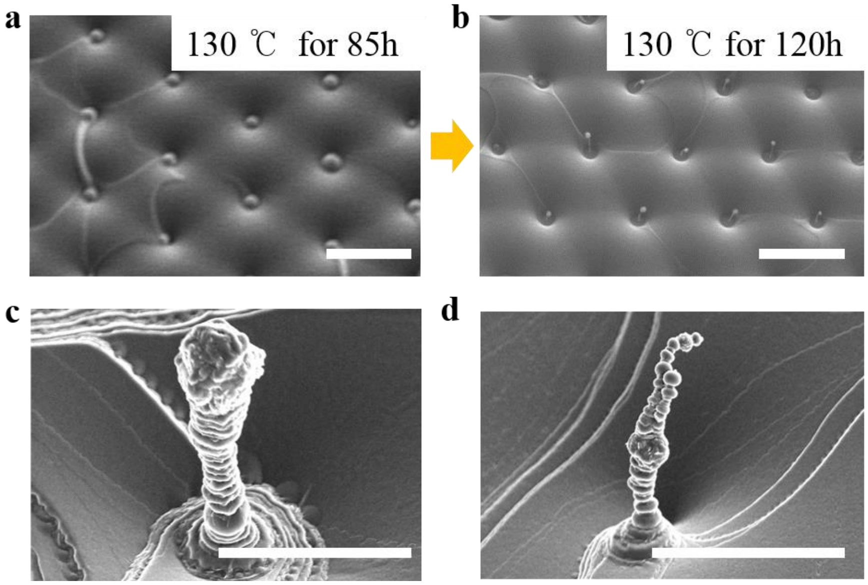

Supplementary Fig. 1. (a,b) SEM images of Udumbara structures formed by thermal annealing at low temperature (130℃). (c,d) SEM images of Udumbara morphologies of Y003 emerging during thermal annealing at 130℃. All scale bars are 10μm.

Supplementary Fig. 2.

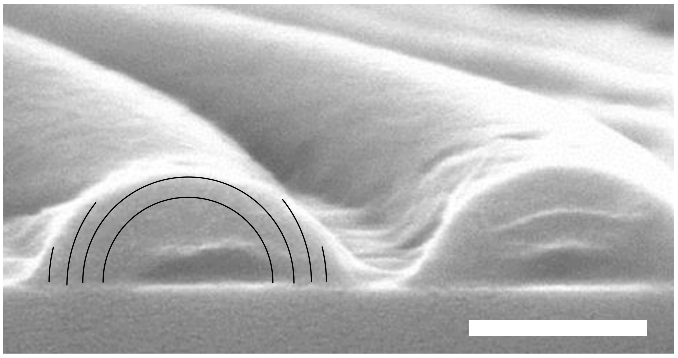

Supplementary Fig. 2. The cross sectional SEM image of rings within the TFCD base formed by sintering at 160 ℃ for 40 min. The sample was cut by glass cutter at room temperature. The shape is not exactly semitoroidal, which can be related either to modified surface anchoring or to the presence of incomplete smectic layers, as shown schematically on the left hand side. The scale bar is 200 nm.

Supplementary Fig. 3

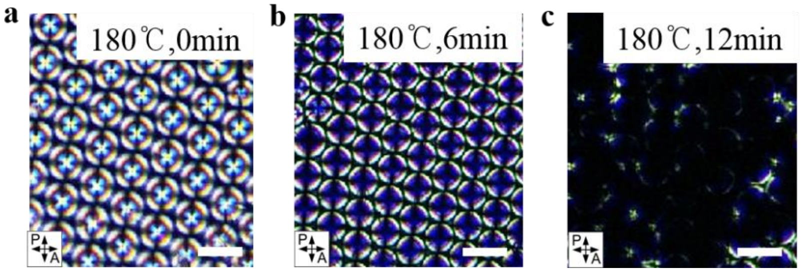

Supplementary Fig. 3. Polarizing optical textures of TFCD film subject to thermal sublimation at 180℃ as a function of time. (a) 0 min, (b) 6min, (c) 12min. All scale bars are 10μm.

Supplementary Fig. 4

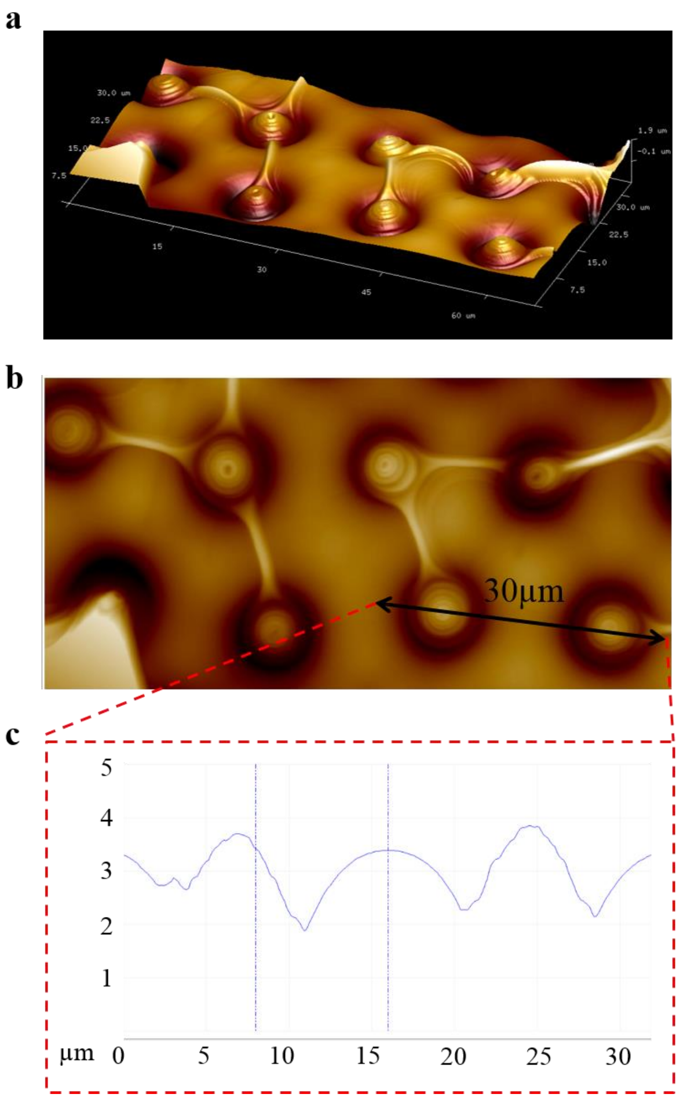

Supplementary Fig. 4. Topographical structure analysis of conical pyramid-like textures formed from TFCD arrays by sintering at 180 ºC for 10 min. The sample was analysed at room temperature.

Supplementary Fig. 5

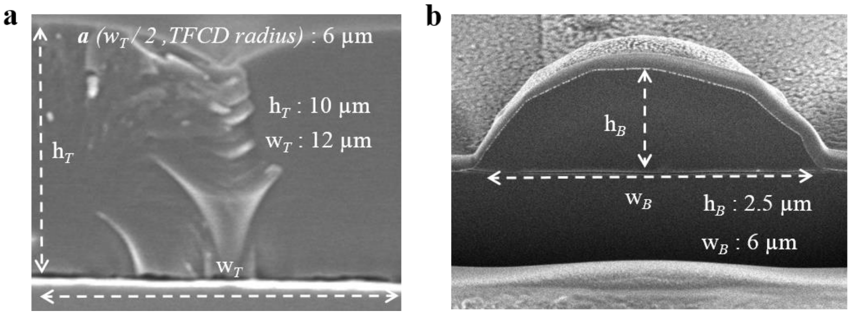

Supplementary Fig. 5. The cross sectional SEM images of the original TFCD and dome structure that occurs after thermal treatment. (a) The volume of TFCD (V_T) is about 1130 μm^3 and the volume of dome structure (V_B) ~ 113 μm^3, showing the evaporated volume ratio (V_T – V_B) / V_T = 0.9.

Supplementary Fig. 6

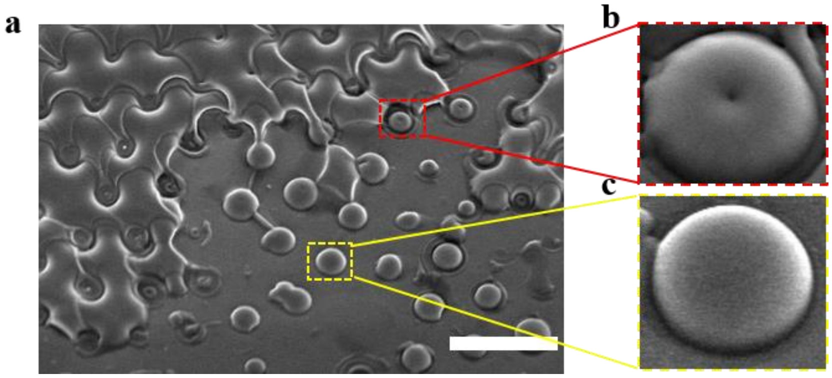

Supplementary Fig. 6. (a) SEM images of SmA film sintered at 190 ºC (the scale bar is 10 μm) with **(b)** toroidal and **(c)** spherical shape replacing the original TFCDs.

Supplementary Fig. 7

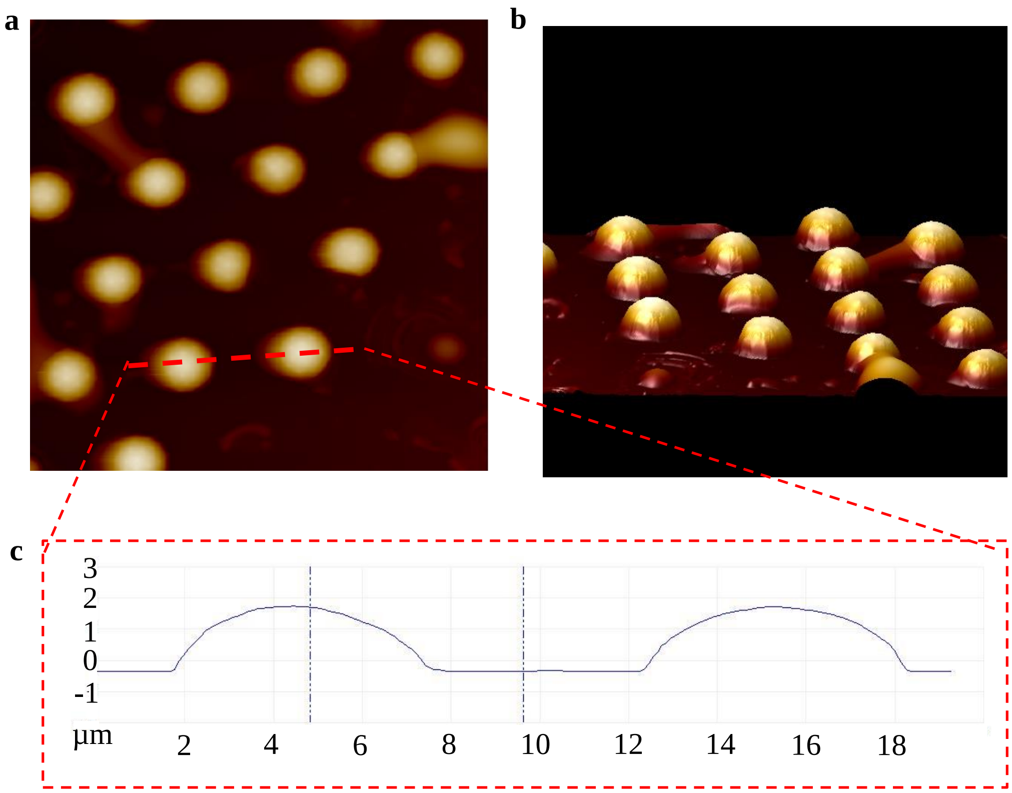

Supplementary Fig. 7. AFM textures of dome structures formed from TFCD by sintering at 190 ºC for 5 min. The measurements are performed at room temperature.

Supplementary Fig. 8

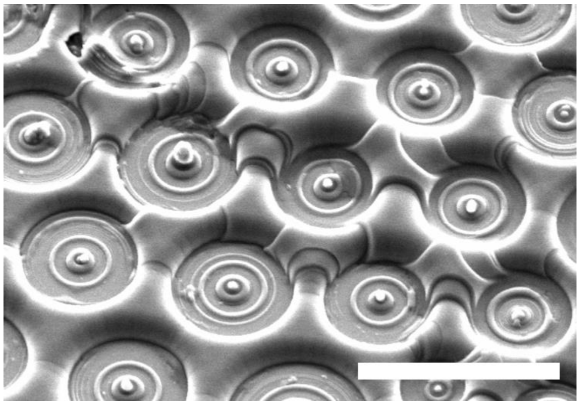

Supplementary Fig. 8. SEM texture of a concentric rings structure formed from an Udumbara structure after a prolonged 200h sintering of the smectic film at 130 ºC. The Udumbara stalk leaves a small piece of the material at the substrate. The scale bar is 20 µm.

Supplementary Fig. 9

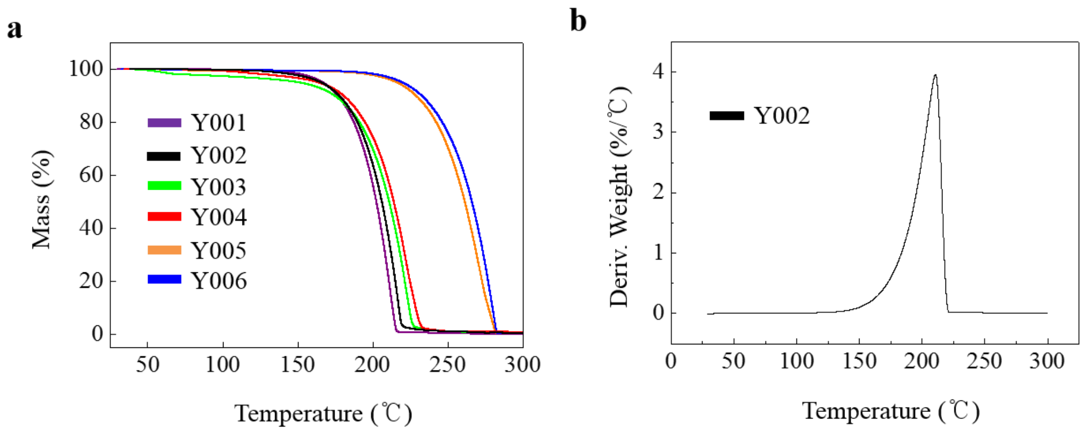

Supplementary Fig. 9. (a) Thermogravimetry analysis (TGA) plots for Y001-Y006 as a function of temperature during heating (heating rate: 1°C/min) shows that sublimation of Y001-Y004 occurred before the material reached their respective isotropic temperatures; Y005, Y006 show very weak sublimation in the SmA phase. (b) The derivative weight percentage of Y002 as a function of temperature presents the sublimation rate.

Supplementary Table 1

	Cooling						
**Y001**	I	207	SmA	146	SmE	69	Cr
**Y002**	I	188	SmA	111	SmE	57	Cr
**Y003**	I	163	SmA	63	SmE	48	Cr
**Y004**	I	154	SmA	59	SmE	42	Cr
**Y005**	I	158	SmA	68	SmE	51	Cr
**Y006**	I	149	SmA	69	SmE	56	Cr

Supplementary Table 1. Phase transition temperatures of the materials during cooling with the rate 5℃min^{-1} (Cr=crystal, SmE=smetic E, SmA=smectic A, I=isotropic phase).
